# Identifying patient and provider determinants of primary care experiences and outcomes for persons with chronic conditions: a multilevel analysis of a nation-wide survey in Norway

**DOI:** 10.1093/fampra/cmag017

**Published:** 2026-04-09

**Authors:** Øyvind Bjertnæs, Mieke Rijken, Ian Porter, Rebecka M Norman, Ingeborg S Sjetne, Hilde H Iversen, Guri Rørtveit, Jose M Valderas

**Affiliations:** Department of Health Services Research, Division for Health Services, Norwegian Institute of Public Health, Oslo 0473, Norway; Primary Care Research Program at NIVEL, Netherlands Institute for Health Services Research (NIVEL), Utrecht, Netherlands; Department of Health and Social Management, University of Eastern Finland, Kuopio, Finland; Department of Health & Community Sciences, Exeter Collaboration for Academic Primary Care (APEx), University of Exeter, Exeter, United Kingdom; Department of Health Services Research, Division for Health Services, Norwegian Institute of Public Health, Oslo 0473, Norway; Department of Health Services Research, Division for Health Services, Norwegian Institute of Public Health, Oslo 0473, Norway; Department of Health Services Research, Division for Health Services, Norwegian Institute of Public Health, Oslo 0473, Norway; Norwegian Institute of Public Health, Oslo 0473, Norway; Department of Health & Community Sciences, Exeter Collaboration for Academic Primary Care (APEx), University of Exeter, Exeter, United Kingdom; Department of Family Medicine, National University Health System, Level 9, Singapore, Singapore; Centre for Research in Health Systems Performance, National University of Singapore, Singapore

**Keywords:** general practice, general practitioner, health care surveys, patient-centered outcomes research, continuity of care, patient satisfaction, physician-patient relations, quality improvement

## Abstract

**Background:**

We aimed to: (i) examine to what extent care experiences and perceived outcomes among persons with chronic conditions depend on the GPs they are registered with and (ii) map the associations between patient and GP-level determinants of patient-reported experience and outcome measures.

**Methods:**

Cross-sectional general practice nested survey of patients (*n* = 6691). We estimated the amount of variation at the GP level and conducted multilevel regression analyses to test hypothesized associations between potential predictors (mostly registry-based) and the six Patient-Reported Experience Measures and five Patient-Reported Outcome Measures as dependent variables.

**Results:**

The ICC for four of six PREMs varied from 0.035 to 0.091, while the ICCs for the PROMs varied from 0.014 to 0.022. The main predictors of patients’ care experiences were health literacy (*P* < 0.001 for all measures) and the number of years on the GP list (*P* < 0.001 for five of six measures). The main predictors for patient-reported outcomes were the number of chronic conditions (*P* < 0.001 for all), income (*P* < 0.001 for all indicators for low income compared to high), health literacy (*P* < 0.001 for all) and the number of consultations in the last 24 months (*P* < 0.001 for all), and the number of years on the GP list was also significantly associated with all PROMs.

**Conclusions:**

Most PREMs demonstrated substantial variation between GPs, warranting GP-level initiatives to improve care experiences for persons with chronic conditions. Health literacy and continuity of care were important determinants of patient-reported experiences and outcomes, demonstrating the importance of these factors for health system performance.

Key messages

- Care experiences and outcomes from the perspective of primary care service users with chronic conditions are not well understood.
- Patient-reported experiences among persons with chronic conditions varied substantially between general practitioners.
- Higher health literacy and better continuity (number of years with the same GP) were systematically and positively associated with patient-reported experiences and outcomes.
- Poorer care experiences or reported outcomes were found for persons with low education or low income; women; and persons born in Eastern Europe, Asia, Africa, and South America.

## Introduction

Chronic conditions and multimorbidity present significant and growing challenges for health systems worldwide, with negative consequences for mortality, quality of life, the ability to work, and healthcare costs [1,2]. In many countries, a considerable proportion of primary care consultations involve individuals with chronic conditions. However, little information is available about how healthcare systems and services perform from the perspective of people living with chronic conditions [3]. In addition, information is lacking on how patient and care provider characteristics are associated with patient-reported care experiences and outcomes. Previous research has assessed patient and organizational factors associated with patient experiences with general practice, but without a specific focus on care for patients with chronic conditions [4] and without assessing patient-reported outcomes like self-reported health and quality of life [5].

The International Survey of People Living with Chronic Conditions (PaRIS) is an ambitious and innovative OECD initiative that integrates a provider survey with a large-scale patient survey across 19 countries [6]. The PaRIS conceptual framework views patient-reported experiences and outcomes as interconnected, multidimensional dependent outcomes, potentially influenced by a range of factors at the patient, provider, and system levels [7]. However, OECDs flagship report from the first PaRIS survey cycle showed that nine out of ten key patient-reported indicators only had 1–3% variation at the practice level [6], thus explaining only a small proportion of the variation on these indicators. As the size of the variation across providers is often interpreted as a signal for improvement potential [8], this could indicate less relevance of practice-level initiatives to improve results.

Norway is one of the participating countries in PaRIS. Norway has a regular general practitioner scheme covering almost the whole population. Most GPs are self-employed and have responsibility for their own patient list. Thus, patients were sampled from individual GPs in Norway, not from group practices with shared patient lists as was the case in many other countries in PaRIS. For countries with an individual-level organization model, such as the Norwegian regular GP scheme, the practice-level approach is less relevant. We expect that a GP-level approach will yield more provider-level variation than at practice level, at least for patient experiences, as previous research has shown substantial variation between individual physicians within practices [9,10]. Furthermore, access to a national registry gave accurate and complete data for additional registered patient characteristics as well as GP/practice characteristics that were not available in the international PaRIS dataset, and could serve as predictors of PREMs and PROMs at the patient and provider level.

The objectives of our study were to (i) examine to what extent care experiences and perceived outcomes among persons with chronic conditions depend on the GPs they are registered with and (ii) map the associations between a range of potential patient and GP-level determinants of PREMs and PROMs, using both survey data and registry data.

## Methods

### Setting

All residents in Norway are entitled to a regular GP, and around 99% of the population is part of the regular GP scheme [11]. The GPs have a list of patients for whom they have medical responsibility. GPs are gatekeepers for the national insurance scheme, and patients are referred by a GP to specialized medical care when needed. The GP practices are, in general, small units most often with four or more GPs, of which around 80% of GPs being self-employed [12].

### Population and sampling

Norway participated in the international PaRIS survey [6]. The eligible patient population was community-dwelling patients aged 45 years or older with at least one consultation with a primary care provider in the 6 months prior to the survey [6]. In Norway, we used the national GP register as the sampling frame and randomly selected 250 individual GPs from all GPs in Norway (approximately 5200 at the time of measurement). We did not stratify GPs but selected a random sample of GPs from the national sampling frame. The goal was to obtain responses from 100 GPs and 75 patients for each responding GP, which was OECD’s sample size targets for Norway [6]. The patient sample was recruited from GPs who responded to the primary care practice questionnaire, the PaRIS-PCPQ [13]. We received valid responses from 118 GPs in Norway and randomly selected 175 patients from each of these using the eligibility criteria described above. To secure the target of 75 patient responses for each GP, 175 patients were randomly selected for each GP given an estimated patient response rate around 45% (estimate based on a pilot survey in Norway).

### Data collection

The survey invitation was sent to the included patients in May 2023 (*n* = 19 637), using the national digital portal Helsenorge.no. The invitation included a link and a unique ID validation code to the digital questionnaire. We sent up to three SMS reminders to non-respondents in the digital group. Patients not registered for communication through the national portal (12.3%) were sent a postal invitation letter and up to one postal reminder, both including a pen-and-paper questionnaire in Norwegian.

### Registry data

We obtained background data about GPs from the national GP register, while background patient data was collected from the GP register, The Norwegian Control and Payment of Health Reimbursements Database and Statistics Norway (variable lists in Supplementary Appendix S1).

## Questionnaire

The conceptual model for the PaRIS survey has been described elsewhere [7]. The PaRIS patient questionnaire (PARIS-PQ) consisted of 118 items, mainly based on existing validated Patient-Reported Experience Measures (PREMs), Patient-Reported Outcome Measures (PROMs) and potential predictors like health and health care capabilities, health behaviors, and individual and sociodemographic factors. In Norway, we also included a five-item short version of the GP scale of the Patient Experiences with GP Questionnaire, the PEQ-GP [14].

The source patient questionnaire in English is provided as Supplementary Appendix S2.

### Outcome measures

We selected the ten key PaRIS outcome measures [6]. PREMs: (i) confidence in self-management (adapted from the Person-Centered Coordinated Care Experiences Questionnaire—P3CEQ; [15]); (ii) experienced care coordination (adapted five-item subscale of the P3CEQ; [15]); (iii) experienced person-centered care (adapted eight-item subscale of the P3CEQ; [15]); (iv) experienced quality (single item, adapted for PaRIS from the Commonwealth Fund International Health Policy Survey; [16]); (v) trust in the healthcare system (single item, based on OECD Guidelines on measuring trust and similar to questions in selected national surveys).

PROMs: (i) physical health (four-item scale of PROMIS Global scale; [17]); (ii) mental health (four-item scale of PROMIS Global scale; [17]); (iii) social functioning (single item from PROMIS Global scale; [17]); (iv) well-being—(WHO five-item Wellbeing Index; [18]); (v) general health (single item from PROMIS Global scale, [17]). Finally, we included the five-item GP scale from the generic PEQ-GP instrument [14].

### Predictors

We selected predictors based on previous research [5,6, 19] and available data, using survey data and registry data about patients and GPs as potential predictors (see Supplementary Appendix S1). Considering the study objectives and the broadness of outcomes, we were inclusive in the selection process to avoid excluding potentially important predictors in multilevel analysis. Chronic condition(s) and health literacy were based on self-report (Supplementary Appendix S2), the latter represented by a scale with two items from the PaRIS survey, which had been constructed from the Porter-Novelli scale [20].

### Statistical analysis

Descriptive statistics were generated for patient and provider background characteristics and the 11 outcome measures. We ran empty multilevel models (patient and GP levels) to estimate the intraclass correlation coefficient (ICC), i.e. the provider-level variation divided by the total variation. Thresholds for defining low variability are uncommon (Fung *et al.* [8]), but based on the ICC and the number of valid responses we calculated the provider-level reliability for each outcome. Provider-level reliability is the proportion of the variance of GP-level mean scores that is due to actual differences between GPs, with reliability greater than 0.70 indicating acceptable reliability [21,22]. We hypothesized that the clustering effect would be largest for GP experiences which are GP specific, followed by experienced quality/coordination/person centeredness which are somewhat less specific. Confidence in self-management is a capability [7], while trust in the health system is much broader than the GP, hence we expected both to have small cluster effects. For PROMs, the level of clustering was expected to be more homogenous as the constructs are a lot more distal than PREMs, hence less direct impact.

We conducted multilevel analysis of the association between patient- and GP-level predictors and the PREMs and PROMs. To assess patient-level and GP-level predictors for the 11 measures, we ran multilevel linear regression models with patient variables as fixed predictors at level 1, providers as a random intercept and provider-level variables as fixed effects at level 2. We report standardized coefficients in the main tables and unstandardized coefficients in Supplementary Appendix S3.

All statistical analyses were conducted with R (version 4.3.0).

## Results

### Patient and GP characteristics

Of the 19 637 approached patients, 8683 responded to the questionnaire (44.2%), of which 6691 reported one or more chronic conditions (our study sample). The majority of persons with chronic conditions were women (54.8%), while 55–69-year-olds comprised the largest age group with 43.8% of respondents (Table 1). Of the 118 GPs, 54.2% were men, 66.1% were 50 years or younger, and 60.2% were a specialist in general practice (Table 1).

**Table 1:** Sample characteristics^a^.

	Respondents/participants	
Variables	Frequency	%
**Patient**		
Sex		
Women	3667	54.8
Men	3024	45.2
Age group		
45–54	1280	19.1
55–69	2929	43.8
70–79	1685	25.2
80+	797	11.9
Education		
Low	1079	16.1
Medium	3133	46.8
High	2441	36.5
Unknown	38	0.6
Income		
Low	1629	24.3
Medium	2092	31.3
High	1701	25.4
Prefer not to say	801	12.0
Don’t know	353	5.3
Birth country/region		
Norway	6193	92.6
Nordic, except Norway	126	1.9
Western Europe, North America, or Oceania	146	2.2
Eastern Europe	86	1.3
Asia, Africa, or South America	139	2.1
Number of self-reported chronic conditions		
1	2969	44.4
2	2088	31.2
3+	1634	24.4
Number of unique diagnoses last 24 months		
1–3	1616	24.2
4–6	2473	37.0
7 or more	2602	38.9
Municipality type		
Large city	1113	16.6
Town or suburb	2962	44.3
Rural	2540	38.0
Don’t know	37	0.6
Missing	39	0.6
Number of GP consultations last 24 months		
1–9	2047	30.6
10–19	2430	36.3
20+	2214	33.1
Number of years on GP list		
0–1	1279	19.1
2–5	1895	28.3
6–15	2027	30.3
16+	1490	22.3
**Primary care provider**		
GP sex		
Women	54	45.8
Men	64	54.2
GP age, years		
40 or younger	38	32.2
41–50	40	33.9
51–60	22	18.6
60+	18	15.3
GP specialist		
Yes	71	60.2
No	47	39.8
Number of years as GP		
0–5	32	27.1
6–10	20	16.9
11–19	34	28.8
20+	32	27.1
List length		
0–900	42	35.6
901–1100	30	25.4
1101 or more	46	39.0
Available spots on patient list		
Yes	64	54.2
No	54	45.8
Use of locum doctors last year		
No use of locum doctors	80	67.8
Locum doctors up to 50%	31	26.3
Locum doctors 50% or more	7	5.9
Number of GPs in GP practice		
1–3	21	17.8
4–5	48	40.7
6 or more	49	41.5
Employment		
Self-employed	97	82.2
Municipality	21	17.8

^a^Registry data, except number of chronic conditions, income and living area which were self-reported by patients. Source patient questionnaire in English: Supplementary Appendix S1.

### Reliability and cluster effects

The GP-level reliability was satisfactory for experienced care coordination (reliability: 0.67), person-centered care (reliability: 0.74), experienced quality (reliability: 0.83), and GP experience (reliability: 0.83) (Table 2). For the two PREMs with highest GP-level reliability (>0.80), the mean GP-level scores varied from 52.1 to 86.6 (SD: 6.3, GP experiences) and 40.0 to 86.4 (SD: 8.3, experienced quality). For PROMs the provider-level reliability varied from 0.44 on social functioning to 0.56 on well-being.

**Table 2:** Descriptive statistics, provider-level variation, and reliability for patient-reported experiences (PREMs) and patient-reported outcomes (PROMs)^a^.

	Individual level			Provider level^b^	
	*n*	Mean	SD	ICC	Reliability
PREMs					
1. Confidence to self-manage (item)	6602	50.8	26.6	0.007	0.28
2. Experienced care coordination (scale)	6680	7.6	3.8	0.035	0.67
3. Person-centered care (scale)	5053	17.0	4.1	0.061	0.74
4. Experienced quality (item)	6477	71.9	24.2	0.084	0.83
5. Trust in health system (item)	6637	70.4	21.5	0.015	0.46
6. GP experiences (scale)	5909	75.3	18.1	0.091	0.83
PROMs					
7. Physical health (scale)	6588	46.6	8.4	0.021	0.54
8. Mental health (scale)	6603	47.0	8.1	0.018	0.51
9. Social functioning (item)	6654	58.2	26.0	0.014	0.44
10. Well-being (scale)	6598	63.0	20.1	0.022	0.56
11. General health (item)	6665	43.2	22.5	0.020	0.54

^a^Indicators 1, 4–6 and 9–11 scored 0–100 where 100 is the best possible score. Indicator 2 ranges from 0–15 where 15 is the best, while indicator 3 ranges from 0–24 where 24 is the best score. Indicator 7–8 transformed to t-score metric in which 50 is the mean and 10 the standard deviation of the PROMIS reference population.

^b^Intraclass correlation coefficient (ICC): the variation between providers divided by the total variation. Reliability: the proportion of the variance at the provider level that is due to true differences between providers.

Four of the PREMs had substantial variation at the GP level: experienced care coordination (ICC: 0.035), experienced person-centered care (ICC: 0.061), experienced quality (ICC: 0.084), and GP experiences (ICC: 0.091). The ICCs for the PROMs were low (0.01–0.02).

### Determinants of PREMs

Health literacy was positively associated with all PREMs, with beta values ranging from 0.159 for experienced coordination to 0.375 for confidence in self-management (*P* < 0.001 for all, Table 3). The number of years on the GP list and age were positively associated with five of six PREMs (*P* < 0.001 for five of six measures), with higher beta values for years on the GP list than age on most measures. Sex and the number of chronic conditions were significantly associated with five PREMs, with four showing better care experiences of men and patients with fewer chronic conditions. The exceptions were that having more chronic conditions was associated with better experienced care coordination (*P* < 0.001), while women reported more confidence in self-management than men (*P* < 0.001). High educated patients reported worse care experiences on four PREMs compared to low educated patients (*P* < 0.05) and on three PREMs compared to patients with a medium-level education (*P* < 0.05).

**Table 3:** Multilevel regressions with patient-reported experience measures (PREMs) as dependent variables^a,b^.

	Confidence to self-manage		Experienced care coordination		Experienced person-centred care		Experienced quality		Trust in health system		GP experiences	
Independent variables	Beta	*P* value	Beta	*P* value	Beta	*P* value	Beta	*P* value	Beta	*P* value	Beta	*P* value
**Patients**												
Age	−0.019	0.157	**0**.**073**	**<0**.**001**	**0**.**098**	**<0**.**001**	**0**.**042**	**0**.**003**	**0**.**075**	**<0**.**001**	**0**.**030**	**0**.**045**
Women (vs men)	**0**.**105**	**<0**.**001**	**−0**.**158**	**<0**.**001**	−0.020	0.508	**−0**.**108**	**<0**.**001**	**−0**.**101**	**<0**.**001**	**−0**.**164**	**<0**.**001**
Education (ref. cat. high):												
Low education	0.084	0.071	**0**.**116**	**0**.**016**	**0**.**133**	**0**.**013**	**0**.**110**	**0**.**023**	0.027	0.578	**0**.**228**	**<0**.**001**
Medium education	0.031	0.268	**0**.**103**	**<0**.**001**	0.057	0.072	**0**.**061**	**0**.**036**	**−0**.**058**	**0**.**044**	**0**.**090**	**0**.**003**
Income (ref. cat. high)												
Low income	−0.067	0.084	−0.010	0.800	−0.054	0.212	−0.075	0.063	**−0**.**190**	**<0**.**001**	−0.060	0.145
Medium income	−0.010	0.764	**0**.**073**	**0**.**032**	0.066	0.069	−0.017	0.606	−0.044	0.188	0.017	0.610
Income “prefer not to say”	**0**.**107**	**0**.**029**	−0.026	0.613	−0.011	0.844	−0.072	0.154	**−0**.**205**	**<0**.**001**	0.019	0.724
Income “don't know”	−0.018	0.785	0.078	0.253	0.079	0.313	−0.030	0.665	**−0**.**265**	**<0**.**001**	0.043	0.559
Birth country (ref. cat. Norway)												
Nordic	−0.078	0.383	0.047	0.614	<0.001	0.997	−0.036	0.692	0.077	0.399	0.022	0.810
Western Europe. North America, Oceania	0.022	0.199	0.001	0.956	0.006	0.748	**−0**.**037**	**0**.**040**	**−0**.**039**	**0**.**028**	0.001	0.961
Eastern Europe	−0.053	0.062	0.040	0.165	−0.061	0.065	**−0**.**093**	**0**.**001**	**−0**.**026**	0.359	−0.010	0.724
Asia, Africa, South America	0.037	0.227	0.019	0.539	−0.061	0.102	**−0**.**129**	**<0**.**001**	−0.022	0.476	**−0**.**084**	**0**.**010**
Number of self-reported chronic conditions	**−0**.**085**	**<0**.**001**	**0**.**054**	**<0**.**001**	**−0**.**051**	**<0**.**001**	**−0**.**029**	**0**.**032**	−0.006	0.633	**−0**.**045**	**0**.**001**
Number of unique diagnosis last 24 months	0.018	0.213	**0**.**034**	**0**.**033**	0.012	0.503	**0**.**038**	**0**.**019**	0.000	0.992	**0**.**042**	**0**.**010**
Health literacy	**0**.**375**	**<0**.**001**	**0**.**159**	**<0**.**001**	**0**.**295**	**<0**.**001**	**0**.**194**	**<0**.**001**	**0**.**163**	**<0**.**001**	**0**.**176**	**<0**.**001**
Number of consultations last 24 months	**−0**.**053**	**<0**.**001**	**0**.**064**	**<0**.**001**	−0.017	0.319	−0.026	0.107	**−0**.**070**	**<0**.**001**	−0.002	0.907
Number of years on GP list	0.006	0.733	**0**.**076**	**<0**.**001**	**0**.**152**	**<0**.**001**	**0**.**105**	**<0**.**001**	**0**.**093**	**<0**.**001**	**0**.**120**	**<0**.**001**
Municipality type (ref. cat. rural)												
City	−0.012	0.771	0.056	0.236	0.022	0.674	0.067	0.178	**0**.**095**	**0**.**029**	0.058	0.264
Town or suburb	0.020	0.474	−0.007	0.823	−0.028	0.430	0.043	0.195	0.033	0.275	−0.049	0.161
Living alone (vs not)	0.040	0.173	0.010	0.738	−0.003	0.921	−0.006	0.841	0.056	0.059	0.006	0.844
**Primary care providers**												
Men (vs. women)	−0.005	0.858	−0.077	0.060	−0.077	0.094	−0.054	0.283	**−0**.**068**	**0**.**043**	−0.072	0.204
Age	−0.009	0.707	−0.043	0.242	−0.046	0.265	−0.085	0.063	−0.042	0.158	−0.044	0.392
Non-specialist (vs specialist)	0.015	0.670	−0.035	0.493	−0.067	0.249	−0.091	0.159	−0.004	0.922	−0.134	0.067
Number of years as GP	0.007	0.829	−0.019	0.658	−0.062	0.211	0.024	0.653	−0.015	0.688	−0.011	0.856
Use of locum doctor last 12 months (%)	−0.003	0.842	−0.019	0.313	−0.038	0.073	−0.044	0.063	−0.010	0.509	−0.029	0.267
List length	−0.008	0.653	−0.039	0.106	**−0**.**064**	**0**.**019**	**−0**.**069**	**0**.**021**	−0.011	0.582	**−0**.**087**	**0**.**010**
Number of GPs in GP practice	−0.004	0.788	**−0**.**048**	**0**.**015**	**−0**.**063**	**0**.**004**	**−0**.**058**	**0**.**017**	−0.014	0.382	−0.046	0.086
Municipality employed (vs self-employed)	−0.018	0.679	−0.082	0.202	**−0**.**169**	**0**.**021**	**−0**.**193**	**0**.**016**	0.020	0.698	−0.150	0.094

^a^Multilevel regressions with random intercept and fixed effects at level 1 (patient) and level 2 (GP). Beta: standardized coefficients (Supplementary Appendix S2 reports unstandardized coefficients). Statistically significant estimates in bold.

^b^Confidence to self-manage, experienced quality, GP experiences and trust in health systems rescored 0–100 where 100 is the best possible score. Experienced care coordination range from 0–15 where 15 is the best, while person-centered care range from 0–24 where 24 is the best score.

Three variables at the GP level were negatively associated with patients’ care experiences: list length (*P* < 0.05 for three PREMs), practice size (*P* < 0.05 for three PREMs), and being employed by the municipality (*P* < 0.05 for two PREMs).

### Determinants of PROMs

The most important predictors for PROMs (Table 4) were income (*P* < 0.001 for all indicators for low income compared to high; beta values ranging from −0.256 for physical health to −0.425 for mental health), the number of chronic conditions (*P* < 0.001 for all; beta values ranging from −0.143 for mental health to −0.266 for physical health), the number of consultations the last 24 months (*P* < 0.001 for all; beta values ranging from −0.205 for mental health to −0.249 for physical health), and health literacy (*P* < 0.001 for all; beta values ranging from 0.137 for general health to 0.203 for mental health) . Other socio-demographic variables associated with PROMs were age (better scores with higher age, *P* < 0.001 for all), sex (women had poorer scores on two measures, *P* < 0.001), education (poorer scores for low education than high education on three measures, *P* < 0.01), and birth country (Eastern Europe scored poorer than Norwegian-born on all measures, *P* < 0.05; Asia/Africa/South America scored poorer on three, *P* < 0.01). The number of years with the same GP was positively and significantly associated with all PROMs (*P* < 0.001), while two variables at the GP level were negatively associated with PROMs: the use of locum doctors the last 12 months (*P* < 0.05 for all indicators) and the number of years as GP (*P* < 0.05 for three indicators).

**Table 4:** Multilevel regressions with patient-reported outcome measures (PROMs) as dependent variables^a,b^.

	Physical health		Mental health		Social functioning		Well-being		General health	
Independent variables	Beta	*P* value	Beta	*P* value	Beta	*P* value	Beta	*P* value	Beta	*P* value
**Patients**										
Age	**0**.**067**	**<0**.**001**	**0**.**174**	**<0**.**001**	**0**.**048**	**<0**.**001**	**0**.**252**	**<0**.**001**	**0**.**067**	**<0**.**001**
Women (vs men)	**−0**.**151**	**<0**.**001**	−0.037	0.140	−0.016	0.522	**−0**.**144**	**<0**.**001**	−0.021	0.383
Education (ref. cat. high)										
Low education	**−0**.**200**	**<0**.**001**	−0.044	0.333	**−0**.**156**	**0**.**001**	0.034	0.441	**−0**.**116**	**0**.**008**
Medium education	**−0**.**094**	**<0**.**001**	−0.012	0.652	−0.032	0.232	0.045	0.087	**−0**.**104**	**<0**.**001**
Income (ref. cat. High)										
Low income	**−0**.**256**	**<0**.**001**	**−0**.**425**	**<0**.**001**	**−0**.**359**	**<0**.**001**	**−0**.**294**	**<0**.**001**	**−0**.**302**	**<0**.**001**
Medium income	**−0**.**084**	**0**.**004**	**−0**.**178**	**<0**.**001**	**−0**.**132**	**<0**.**001**	**−0**.**081**	**0**.**009**	**−0**.**108**	**<0**.**001**
Prefer not to say	**−0**.**177**	**<0**.**001**	**−0**.**271**	**<0**.**001**	**−0**.**193**	**<0**.**001**	**−0**.**080**	0.084	**−0**.**240**	**<0**.**001**
Don't know	**−0**.**285**	**<0**.**001**	**−0**.**374**	**<0**.**001**	**−0**.**300**	**<0**.**001**	**−0**.**142**	**0**.**022**	**−0**.**294**	**<0**.**001**
Birth country (ref. cat. Norway)										
Nordic	−0.126	0.117	−0.020	0.813	0.006	0.946	−0.103	0.218	−0.038	0.653
Western Europe, North America, Oceania	−0.025	0.108	−0.015	0.357	−0.004	0.790	−0.019	0.242	0.014	0.387
Eastern Europe	**−0**.**111**	**<0**.**001**	**−0**.**090**	**0**.**001**	**−0**.**074**	**0**.**005**	**−0**.**081**	**0**.**002**	**−0**.**060**	**0**.**021**
Asia, Africa, South America	**−0**.**136**	**<0**.**001**	**−0**.**093**	**0**.**001**	−0.036	0.218	**−0**.**113**	**<0**.**001**	**−0**.**053**	0.061
Number of self-reported chronic conditions	**−0**.**266**	**<0**.**001**	**−0**.**143**	**<0**.**001**	**−0**.**144**	**<0**.**001**	**−0**.**192**	**<0**.**001**	**−0**.**247**	**<0**.**001**
Number of unique diagnosis last 24 months	**−0**.**047**	**0**.**001**	−0.003	0.811	0.022	0.124	−0.013	0.368	**−0**.**031**	**0**.**028**
Health literacy	**0**.**182**	**<0**.**001**	**0**.**203**	**<0**.**001**	**0**.**192**	**<0**.**001**	**0**.**180**	**<0**.**001**	**0**.**137**	**<0**.**001**
Number of consultations last 24 months	**−0**.**249**	**<0**.**001**	**−0**.**205**	**<0**.**001**	**−0**.**219**	**<0**.**001**	**−0**.**229**	**<0**.**001**	**−0**.**240**	**<0**.**001**
Number of years on GP list	**0**.**090**	**<0**.**001**	**0**.**099**	**<0**.**001**	**0**.**092**	**<0**.**001**	**0**.**101**	**<0**.**001**	**0**.**093**	**<0**.**001**
Municipality type										
City (vs rural)	0.048	0.220	−0.030	0.448	0.069	0.075	−0.043	0.264	0.057	0.148
Town or suburb (vs rural)	0.005	0.848	−0.024	0.394	0.034	0.225	−0.013	0.621	0.032	0.250
Living alone (vs not)	−0.010	0.709	**0**.**056**	**0**.**046**	0.043	0.126	**0**.**060**	**0**.**027**	−0.032	0.239
**Primary care providers**										
Men (vs. women)	−0.033	0.295	0.028	0.322	0.033	0.242	−0.012	0.667	−0.032	0.280
Age	0.017	0.540	0.012	0.631	0.027	0.269	0.019	0.463	0.022	0.404
Non-specialist (vs specialist)	0.034	0.387	0.025	0.479	0.035	0.314	0.026	0.466	−0.011	0.774
Number of years as GP	−0.027	0.420	**−0**.**062**	**0**.**047**	**−0**.**068**	**0**.**027**	**−0**.**065**	**0**.**037**	−0.049	0.127
Use of locus doctor last 12 months (%)	**−0**.**033**	**0**.**026**	**−0**.**040**	**0**.**003**	**−0**.**029**	**0**.**031**	**−0**.**041**	**0**.**002**	**−0**.**032**	**0**.**022**
List length	0.013	0.477	−0.013	0.451	−0.007	0.687	0.002	0.908	0.018	0.308
Number of GPs in GP practice	0.013	0.380	−0.017	0.198	−0.005	0.682	−0.022	0.098	0.010	0.494
Municipality employed (vs self-employed)	−0.038	0.434	0.011	0.801	0.006	0.895	−0.013	0.778	0.008	0.859

^a^Multilevel regressions with random intercept and fixed effects at level 1 (patient) and level 2 (GP). Beta: standardized coefficients (Supplementary Appendix S2 reports unstandardized coefficients). Statistically significant estimates in bold.

^b^Physical and mental health transformed to t-score metric in which 50 is the mean and 10 the standard deviation of the PROMIS reference population. The other indicators are scored 0–100 where 100 is the best possible score.

## Discussion

A substantial part of the variation in primary care patients’ care experiences was related to the GP they receive care from. Health literacy, the number of chronic conditions, age, and the number of years on the GP list were consistent predictors for patients’ care experiences and self-reported outcomes. Several other socio-demographic factors (sex, education, birth country) were weakly or moderately related to patients’ care experiences and self-reported outcomes, while income was strongly related to all patient-reported outcomes and trust in the health system.

OECDs PaRIS flagship report showed that most PREMs and PROMs had little variation at the provider level, with sampling on the practice level for most countries [6]. For countries with an individual-level organization model, such as the Norwegian regular GP scheme, the practice-level approach is less relevant. Our study, restricted to the target group of persons with chronic conditions, identified four PREMs with substantial variation between GPs, all with satisfactory GP-level reliability. Previous research has shown substantial variation between physicians within a practice [9,10], meaning that a practice-level approach will average out differences in the experiences of patients with individual physicians within practices and consequently produce lower ICCs. Thus, our findings were as expected but show the importance of in-depth within-country data and analyses to supplement the international analyses. Our study underlines the relevance and importance of measuring and improving patient experiences at the GP level in Norway, and probably in other countries with an individual-level organization model.

Health literacy was the single most important predictor of variation in the key measures. Health literacy has been connected to health outcomes in previous systematic reviews [23,24], but the association with PREMs is more seldom assessed. We found that health literacy had an especially strong association with two PREMs, namely confidence in self-management and patient-centered care. Confidence in self-management was included as a PREM in the OECD flagship report [6], but according to the conceptual model this is not a PREM but a health and health care capability construct [7]. The data empirically supported the conceptual model by showing a pattern of association quite different from all other PREMs, including no association with any of the provider-level variables, while it showed the highest degree of association with the other capability measure, health literacy. Incidentally it is not surprising that person-centeredness also has also an association with literacy as all these capabilities measures (literacy, confidence, and activation) are a prerequisite to effectively implementing a person-centered approach. The most important risk factor for low health literacy is low education [25], which means that less educated persons with chronic conditions are an important target group for initiatives to strengthen health literacy, confidence in self-management and person-centered care. We also found poorer results for persons with low income, for women, younger persons and some birth country groups, which are additional target groups for interventions.

Continuity of care was linked to patients experiencing better care and better outcomes, with the number of years on the GP list positively related to both, and less use of locum doctor positively related to patients’ reported outcomes. Previous research has identified a connection between continuity and a range of positive outcomes [26-28], and GP continuity was the key predictor for patient-perceived accessibility to general practice in a recent Norwegian study [29]. We are not aware of previous nationwide studies among patients in general practice showing a consistent association between continuity of care and broad PROMs related to physical health, mental health, social functioning, well-being, and general health. Thus, our study adds value to the literature on the importance of continuity in primary care for persons with chronic conditions [26-29]. Several other GP-level variables were associated with two or more measures including the number of GPs in the practice, list length, employment type, and the number of years working as GP. These are potential GP and practice-level factors that could be leveraged in efforts to improve care experiences and outcomes for persons with chronic conditions, but we underline that these were less important than the most important patient-level predictors.

### Limitations

Less than half of the GPs participated and the response rate for patients was 44%. This opens the possibility for selection bias and non-response bias. Compared to the other 18 countries participating in the OECD PaRIS survey, Norway had a high response rate for both patients and providers [6]. E.g. the patient response rate varied from 6% in Romania to 47% in Spain, with Norway having the second highest response rate for patients (44%), only slightly below Spain [6]. We used two interconnected outcomes in our study (PREMs, PROMs), but without assessing how they are connected. The conceptual and empirical broadness of the PaRIS survey facilitates the assessment of the interconnection between these constructs [7], which should be an important topic for further research. We aimed to map the associations between a range of potential patient and GP-level determinants of PREMs and PROMs, but associations do not imply causality. The complex interaction between predictors, PREMs, and PROMs should be assessed in future research. The generalizability to countries with a different structure and organization of primary care is questionable, especially the cluster effects and provider-level predictors. We recommend further research in other countries using physician-level sampling or other approaches to conduct disaggregated analyses.

### Implications

Chronic conditions and multimorbidity have negative consequences for quality of life, the ability to work and healthcare costs [2, 30,31]. Our findings stress the necessity for novel primary care models to empower and better support the growing number of persons living with chronic conditions [32,33], extending beyond traditional healthcare's single disease focus. There is a need for more person-centered primary care focusing on the needs and preferences of persons with chronic conditions, including health literacy, self-management support, continuity, and coordinated care.

The large differences in care experiences and outcomes between patients with high and limited health literacy, and the large differences in trust in the health system and self-reported outcomes between high- and low-income groups underline the importance of tailoring primary care services to disadvantaged groups. While health inequalities are well documented [34], our study showed systematic inequality across a broad set of performance dimensions for primary care users. To ensure improvements for all it is essential that improvement efforts and research measure and monitor not only overall effects, but effects on inequality as well [35,36].

## Conclusions

Patients’ care experiences depended for a substantial part on the GP they receive care from, while health literacy and relationship continuity were the most important predictors of patients’ care experiences and outcomes. A shortage of primary care physicians, together with aging populations and a growing number of people with chronic conditions among future users of primary care, makes it essential to develop and implement person-centered, effective, and innovative primary care models. These should promote health literacy and self-management capabilities among people with chronic conditions, while also being responsive to possible inequalities across socio-demographic groups.

## Supplementary Material

cmag017_Supplementary_Data

## Data Availability

Anonymous data is available from the corresponding author on reasonable request.
