# Supplementary material for: Identifying patient and provider determinants of primary care experiences and outcomes for persons with chronic conditions: a multilevel analysis of a nation-wide survey in Norway
Source: Fam Pract. 2026 Apr 9;43(3):cmag017. doi: 10.1093/fampra/cmag017 (PMC13070000; doi:10.1093/fampra/cmag017)
Supplement: cmag017_Supplementary_Data [file cmag017_supplementary_data.zip › Appendix1.pdf]

Registry data about patients: sex, age, country of birth, level of education, number of consultations with the GP in the last 24 months, diagnoses according to the ICPC system, number of unique ICPC diagnoses in the last 24 months, and duration of registration with the current GP.

Registry data about GPs: sex, age, specialist in general practice or community medicine (yes/no), duration of work as GP, list size, available spots on GP list for those without a full patient list, use of locum doctors (temporary substitutes) last year, the number of GPs in the GP practice and whether the GP was self-employed or employed by the municipality.
