# Supplementary material for: Identifying patient and provider determinants of primary care experiences and outcomes for persons with chronic conditions: a multilevel analysis of a nation-wide survey in Norway
Source: Fam Pract. 2026 Apr 9;43(3):cmag017. doi: 10.1093/fampra/cmag017 (PMC13070000; doi:10.1093/fampra/cmag017)
Supplement: cmag017_Supplementary_Data [file cmag017_supplementary_data.zip › Appendix3.pdf]

Appendix 3. Multilevel regressions[a] with Patient-Reported Experiences[b] as dependent variables (unstandardized coefficients)

	Confidence to self-manage		Experienced care coordination		Person-centred care		Experienced quality		Trust in health system		GP experiences	
	B	P value	B	P value	B	P value	B	P value	B	P value	B	P value
**Patient factors**:												
Age	-0.046	0.157	**0.025**	**<0.001**	**0.036**	**<0.001**	**0.092**	**0.003**	**0.144**	**<0.001**	**0.049**	**0.045**
Women (vs men)	**2.803**	**<0.001**	**-0.597**	**<0.001**	-0.081	0.508	**-2.615**	**<0.001**	**-2.170**	**<0.001**	**-2.957**	**<0.001**
Education (ref. cat. high):												
Low education	2.237	0.071	**0.439**	**0.016**	**0.541**	**0.013**	**2.668**	**0.023**	0.570	0.578	**4.117**	**<0.001**
Medium education	0.822	0.268	**0.389**	**<0.001**	0.231	0.072	**1.478**	**0.036**	**-1.244**	**0.044**	**1.616**	**0.003**
Income (ref. cat. high):												
Low income	-1.790	0.084	-0.039	0.800	-0.221	0.212	-1.815	0.063	**-4.071**	**<0.001**	-1.086	0.145
Medium income	-0.260	0.764	**0.276**	**0.032**	0.269	0.069	-0.423	0.606	-0.948	0.188	0.315	0.610
Income "prefer not to say"	**2.853**	**0.029**	-0.097	0.613	-0.046	0.844	-1.752	0.154	**-4.388**	**<0.001**	0.335	0.724
Income "don't know"	-0.481	0.785	0.295	0.253	0.322	0.313	-0.717	0.665	**-5.694**	**<0.001**	0.774	0.559
Birth country (ref. cat. Norway):												
Nordic	-2.064	0.383	0.176	0.614	-0.002	0.997	-0.879	0.692	1.651	0.399	0.400	0.810
Western Europe, North-America, Oceania	0.584	0.199	0.004	0.956	0.025	0.748	**-0.893**	**0.040**	**-0.832**	**0.028**	0.016	0.961
Eastern Europe	-1.398	0.062	0.151	0.165	-0.248	0.065	**-2.256**	**0.001**	-0.558	0.359	-0.187	0.724
Asia, Africa, South-America	0.994	0.227	0.073	0.539	-0.246	0.102	**-3.112**	**<0.001**	-0.476	0.476	**-1.517**	**0.010**
Number of self-reported chronic conditions	**-2.808**	**<0.001**	0.253	**<0.001**	**-0.257**	**<0.001**	**-0.858**	**0.032**	-0.168	0.633	**-1.014**	**0.001**
Number of unique diagnosis last 24 months	0.624	0.213	**0.162**	**0.033**	0.060	0.503	**1.164**	**0.019**	-0.004	0.992	**0.982**	**0.010**
Health literacy	**0.450**	**<0.001**	**0.027**	**<0.001**	**0.054**	**<0.001**	**0.212**	**<0.001**	**0.158**	**<0.001**	**0.143**	**<0.001**
Number of consultations last 24 months	**-0.096**	**<0.001**	**0.016**	**<0.001**	-0.005	0.319	-0.042	0.107	**-0.101**	**<0.001**	-0.002	0.907
Number of years on GP list	0.164	0.733	**0.278**	**<0.001**	**0.597**	**<0.001**	**2.442**	**<0.001**	**1.928**	**<0.001**	**2.097**	**<0.001**
Municipality type (ref. cat. rural):												
City	-0.309	0.771	0.210	0.236	0.088	0.674	1.619	0.178	**2.037**	**0.029**	1.042	0.264

Town or suburb	0.542	0.474	-0.027	0.823	-0.115	0.430	1.045	0.195	0.713	0.275	-0.879	0.161
Living alone	1.053	0.173	0.038	0.738	-0.013	0.921	-0.146	0.841	1.206	0.059	0.111	0.844
**GP/GP office factors:**												
Men (vs. women)	-0.135	0.858	-0.290	0.060	-0.314	0.094	-1.301	0.283	**-1.462**	**0.043**	-1.292	0.204
Age	-0.023	0.707	-0.015	0.242	-0.017	0.265	-0.189	0.063	-0.083	0.158	-0.073	0.392
Non-specialist (vs specialist)	0.405	0.670	-0.134	0.493	-0.275	0.249	-2.194	0.159	-0.089	0.922	-2.415	0.067
Number of years as GP	0.023	0.829	-0.009	0.658	-0.032	0.211	0.074	0.653	-0.040	0.688	-0.025	0.856
Use of locus doctor last 12 months (%)	-0.005	0.842	-0.005	0.313	-0.010	0.073	-0.068	0.063	-0.014	0.509	-0.034	0.267
List length	-0.001	0.653	-0.001	0.106	**-0.001**	**0.019**	**-0.006**	**0.021**	-0.001	0.582	**-0.005**	**0.010**
Number of GPs in GP office	-0.040	0.788	**-0.074**	**0.015**	**-0.106**	**0.004**	**-0.572**	**0.017**	-0.122	0.382	-0.343	0.086
Municipality employed (vs self-employed)	-0.491	0.679	-0.307	0.202	**-0.687**	**0.021**	**-4.675**	**0.016**	0.437	0.698	-2.704	0.094

[a] Multilevel regressions with random intercept and fixed effects at level 1 (patient) and level 2 (GP). Statistically significant estimates in bold. [b] Confidence to self-manage, experienced quality, GP experiences and trust in health systems scored 0-100 where 100 is the best possible score. Experienced care coordination range from 0-15 where 15 is the best, while person-centred care range from 0-24 where 24 is the best score.

Multilevel regressions[a] with Patient-Reported Outcomes[b] as dependent variables (unstandardized coefficients)

	Physical health		Mental health		Social functioning		Well-being		General health	
	B	P value	B	P value	B	P value	B	P value	B	P value
**Patient factors**:										
Age	**0.051**	**<0.001**	**0.126**	**<0.001**	**0.113**	**<0.001**	**0.454**	**<0.001**	**0.134**	**<0.001**
Women (vs men)	**-1.274**	**<0.001**	-0.300	0.140	-0.419	0.522	**-2.899**	**<0.001**	-0.480	0.383
Education (ref. cat. high):										
Low education	**-1.688**	**<0.001**	-0.352	0.333	**-4.053**	**0.001**	0.676	0.441	**-2.611**	**0.008**
Medium education	**-0.792**	**<0.001**	-0.098	0.652	-0.842	0.232	0.903	0.087	**-2.340**	**<0.001**
Income (ref. cat. High):										
Low income	**-2.165**	**<0.001**	**-3.425**	**<0.001**	**-9.336**	**<0.001**	**-5.906**	**<0.001**	**-6.786**	**<0.001**
Medium income	**-0.710**	**0.004**	**-1.438**	**<0.001**	**-3.432**	**<0.001**	**-1.624**	**0.009**	**-2.434**	**<0.001**
Prefer not to say	**-1.496**	**<0.001**	**-2.189**	**<0.001**	**-5.029**	**<0.001**	-1.604	0.084	**-5.395**	**<0.001**
Don't know	**-2.404**	**<0.001**	**-3.017**	**<0.001**	**-7.800**	**<0.001**	**-2.855**	**0.022**	**-6.611**	**<0.001**
Birth country (ref. cat. Norway):										
Nordic born	-1.063	0.117	-0.164	0.813	0.151	0.946	-2.067	0.218	-0.844	0.653
Western Europe, North-America, Oceania	-0.209	0.108	-0.123	0.357	-0.115	0.790	-0.377	0.242	0.313	0.387
Eastern Europe	**-0.935**	**<0.001**	**-0.727**	**0.001**	**-1.932**	**0.005**	**-1.634**	**0.002**	**-1.351**	**0.021**
Asia, Africa, South-America	**-1.152**	**<0.001**	**-0.753**	**0.001**	-0.940	0.218	**-2.271**	**<0.001**	-1.192	0.061
Number of self-reported chronic conditions	**-2.793**	**<0.001**	**-1.436**	**<0.001**	**-4.668**	**<0.001**	**-4.782**	**<0.001**	**-6.894**	**<0.001**
Number of unique diagnosis last 24 months	**-0.505**	**0.001**	-0.035	0.811	0.731	0.124	-0.322	0.368	**-0.886**	**0.028**
Health literacy	**0.070**	**<0.001**	**0.074**	**<0.001**	**0.226**	**<0.001**	**0.163**	**<0.001**	**0.139**	**<0.001**
Number of consultations last 24 months	**-0.142**	**<0.001**	**-0.112**	**<0.001**	**-0.385**	**<0.001**	**-0.311**	**<0.001**	**-0.364**	**<0.001**
Number of years on GP list	**0.735**	**<0.001**	**0.774**	**<0.001**	**2.320**	**<0.001**	**1.951**	**<0.001**	**2.021**	**<0.001**
Municipality type:										
City (vs rural)	0.404	0.220	-0.239	0.448	1.803	0.075	-0.868	0.264	1.281	0.148
Town or suburb (vs rural)	0.044	0.848	-0.191	0.394	0.874	0.225	-0.271	0.621	0.715	0.250
Living alone	-0.082	0.709	0.451	0.046	1.117	0.126	**1.208**	**0.027**	-0.720	0.239
**GP/GP office factors**:										

Men (vs. women)	-0.275	0.295	0.226	0.322	0.849	0.242	-0.245	0.667	-0.722	0.280
Age	0.013	0.540	0.009	0.631	0.065	0.269	0.034	0.463	0.046	0.404
Non-specialist (vs specialist)	0.288	0.387	0.204	0.479	0.918	0.314	0.524	0.466	-0.242	0.774
Number of years as GP	-0.029	0.420	**-0.063**	**0.047**	**-0.226**	**0.027**	**-0.167**	**0.037**	-0.142	0.127
Use of locus doctor last 12 months (%)	**-0.018**	**0.026**	**-0.021**	**0.003**	**-0.048**	**0.031**	**-0.054**	**0.002**	**-0.047**	**0.022**
List length	<0.001	0.477	<0.001	0.451	-0.001	0.687	<0.001	0.908	0.001	0.308
Number of GPs in GP office	0.045	0.380	-0.057	0.198	-0.058	0.682	-0.184	0.098	0.089	0.494
Municipality employed (vs self-employed)	-0.322	0.434	-0.090	0.801	0.150	0.895	-0.252	0.778	0.186	0.859

[a] Multilevel regressions with random intercept and fixed effects at level 1 (patient) and level 2 (GP). B: unstandardized coefficients. Statistically significant estimates in bold. [b] Physical and mental health transformed to t-score metric in which 50 is the mean and 10 the standard deviation of the PROMIS reference population. The other indicators are scored 0-100 where 100 is the best possible score.
